# A simple and cost-effective protocol for the management of anterolateral thigh free-flap donor site

**DOI:** 10.4103/0970-0358.44929

**Published:** 2008

**Authors:** Puneet Tuli, Gautam Biswas, Atul Parashar, Ramesh K. Sharma

**Affiliations:** Department of Plastic Surgery, Postgraduate Institute of Medical Education and Research, Sector 12, Chandigarh, India

Sir,

The perforator-based anterolateral thigh flap was first described by Song *et al.*[1] Minimal donor site morbidity and functional impairment has led various authors to use this flap for a wide variety of defects.[2] The management of the donor site poses a unique problem as a large area of skin (upto 800 cm^2^) can be harvested. Primary closure of the donor site with mobilization of surrounding skin is possible only in a few cases when the size of the defect is less than 8 × 8 cm^2^.[2]

The anterolateral thigh flap is invariably used for reconstructing large defects, where a split skin graft is needed in a majority of the cases for closure of the donor site. Although the harvesting of the split skin graft is routine for a plastic surgeon, it still has its own complications such as pain and infection of the donor site in the early postoperative period with a probability of later hypertrophic scarring. For resurfacing of the flap donor site, a split skin graft is usually harvested from another anatomical region which leads to two wounds at different sites.

In our series of 15 consecutive cases of anterolateral thigh flap we harvested split skin grafts from the medial or posterior aspect of the same thigh. The skin graft donor site was dressed with antibiotic-impregnated tulle dressing and absorbent sponge. The flap donor site was resurfaced with the split skin graft and fixed using a tie-over dressing. The tie-over dressing was removed on the 5^th^ postoperative day without disturbing the split skin donor site tulle dressing. All the cases had total (*n*= 13) or near total (*n*= 2) graft take. The skin graft donor sites healed over 14 to 17 days (mean = 15.8 days). Thus, all the patients had dressing limited to one limb and there was no additional donor site morbidity in any of the cases. Late management included the use of a single compression garment, both for the skin graft as well as for the grafted flap donor sites.

**Figure 1:**
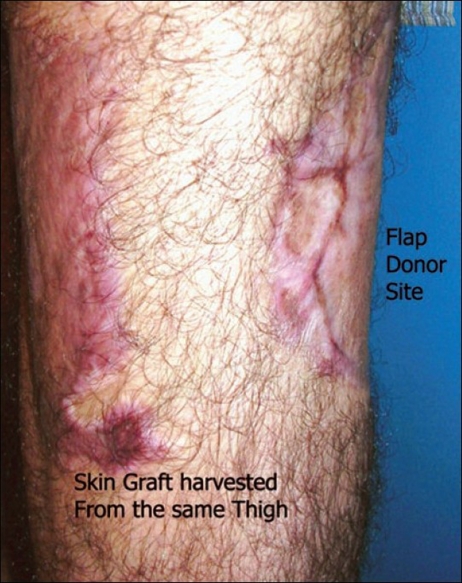
Skin graft for the grafted flap donor sites

To conclude, we suggest that in the management of the donor site of an anterolateral thigh flap, split skin grafts can be harvested from the same thigh without any additional complications. This helps to limit the use of dressing material and time. Postoperative management of the skin graft and flap donor sites becomes simple and cost-effective as both donor and recipient sites can be managed with the same compression garment. In addition, this method helps to restrict the scarring to the same thigh [Figure 1].
